# Multiple Intracranial Meningiomas in Absence of Neurofibromatosis Type 2: A Case Report and Literature Review

**DOI:** 10.7759/cureus.22118

**Published:** 2022-02-11

**Authors:** Jeevesh Mallik, Sharadendu Narayan, Niraj Choudhary

**Affiliations:** 1 Department of Neurosurgery, Tata Main Hospital, Jamshedpur, IND

**Keywords:** tumors, neurofibromatosis type 2, multiple meningiomas, benign, meningiomas

## Abstract

Meningiomas are one of the most common primary intracranial tumors known to exist since pre-historic times. Most of these tumors are benign, sporadic, and solitary. Multiple meningiomas are rare and have mostly been described in patients with neurofibromatosis type 2 (NF2). The presence of multiple lesions poses a unique challenge in strategizing the treatment. We present a rare case of multiple intracranial meningiomas in the absence of NF2, which we treated at Tata Main Hospital, Jamshedpur. The relevant literature has also been discussed.

## Introduction

Meningiomas are tumors derived from the neuroectoderm and arise from arachnoid cap cells [1]. These cells are located at sites of arachnoid granulations on the inner surface of the dura mater. The anatomical incidence of meningiomas roughly corresponds to the distribution of arachnoid villi. In the vast majority, these tumors are solitary. Multiple meningiomas have been seen only in 2.3-8.9% of all patients, most of whom have neurofibromatosis type 2 (NF2).

## Case presentation

A 67-year-old female presented to our outpatient unit with complaints of weakness of the right side of her body for six months. Her weakness was insidious in onset and had progressively increased over the last six months, and at the time of presentation, she was unable to bear the weight of her right leg and was unable to feed herself with her right hand. She also complained of headaches for six months, mostly on her left side, which had increased in severity for one month. In addition, she had had three episodes of seizures which started with a jerky movement of her right hand followed by generalized tonic-clonic seizures.

She had no history of trauma, fever, or bladder/bowel involvement. She had no history of diabetes or hypertension. She was hypothyroid and was on 75 mcg thyroxine. There was no family history of a similar illness.

On examination, she was conscious and oriented with stable vitals. On clinical examination, her recent memory was impaired, with the rest of the higher mental functions being normal. Upon motor examination, the tone was increased in both the right upper and lower limb. She had poor grip in her right hand, grade 3 power around the hip and knee, and Grade 2 power around the right ankle in her right lower limb. The rest of the motor examination was normal. Sensory examination was also normal. All deep tendon reflexes were exaggerated in the right upper and lower limbs. Plantar examination showed an equivocal response. The rest of her neurological examination was normal.

**Figure 1:**
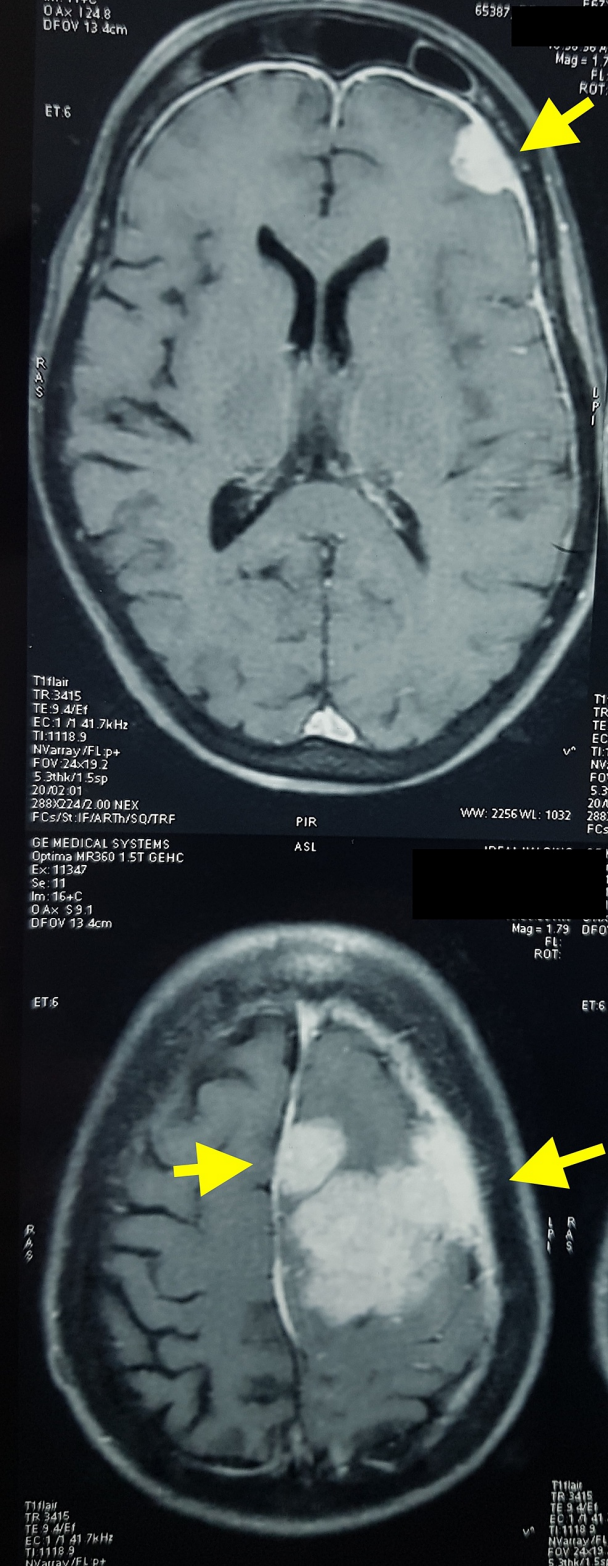
Pre-operative MRI axial images showing multiple meningiomas.

**Figure 2:**
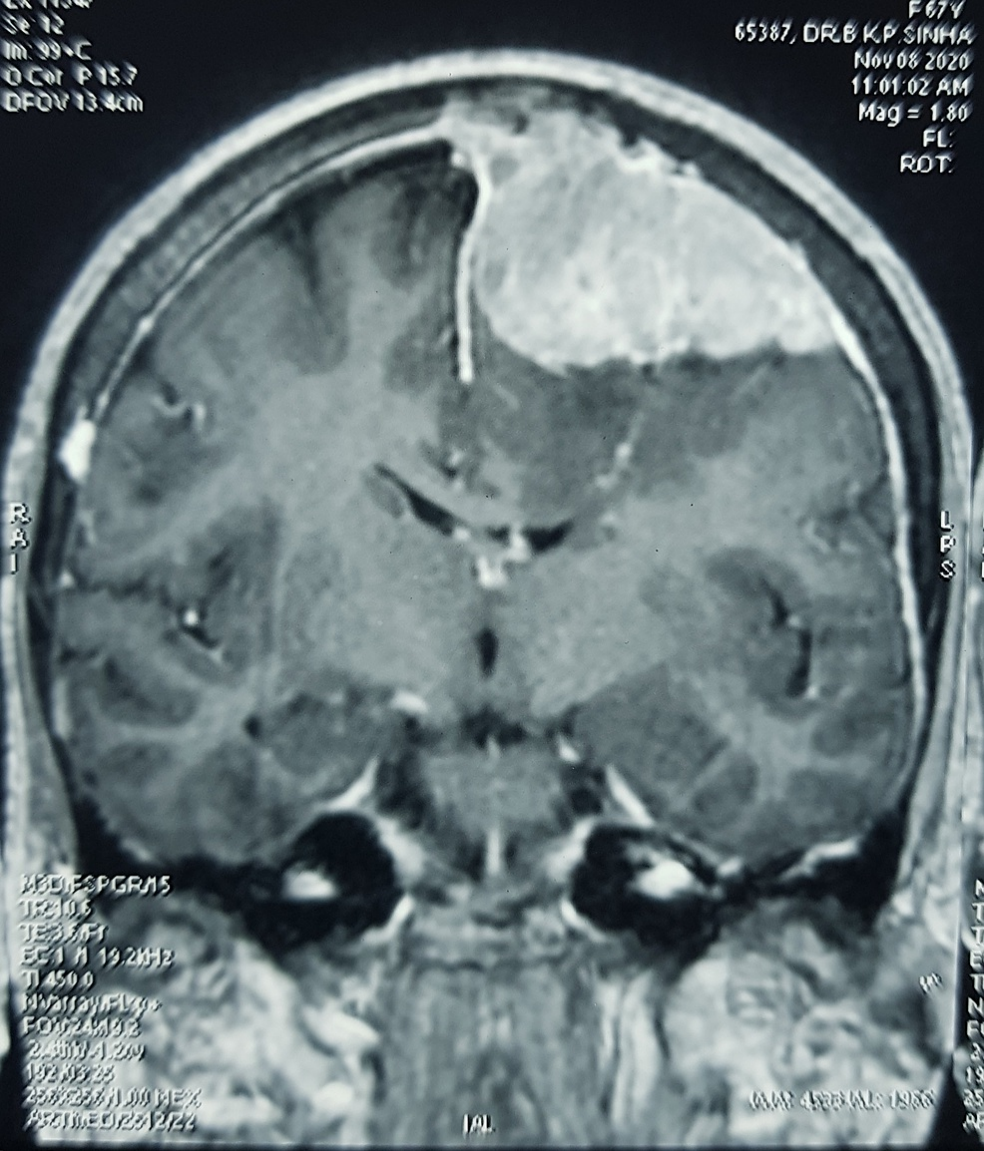
Pre-operative MRI coronal image.

MRI brain was obtained, which showed multiple meningiomas in the left frontal region; three lesions were seen (Figures 1-2). The left frontal convexity and the parafalcine small lesion showed no perilesional edema and were planned for observation. 

**Figure 3:**
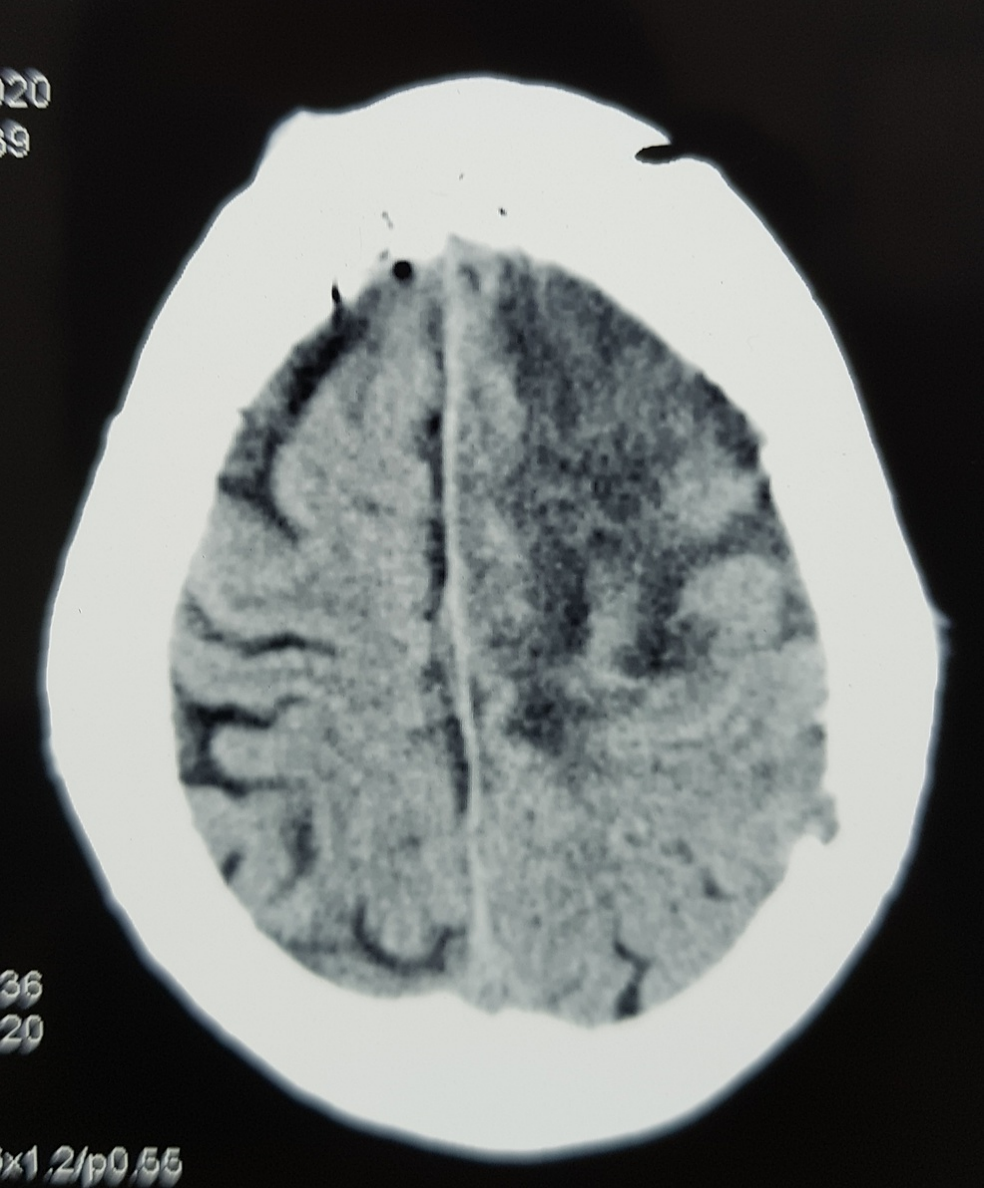
Post-operative CT scan.

**Figure 4:**
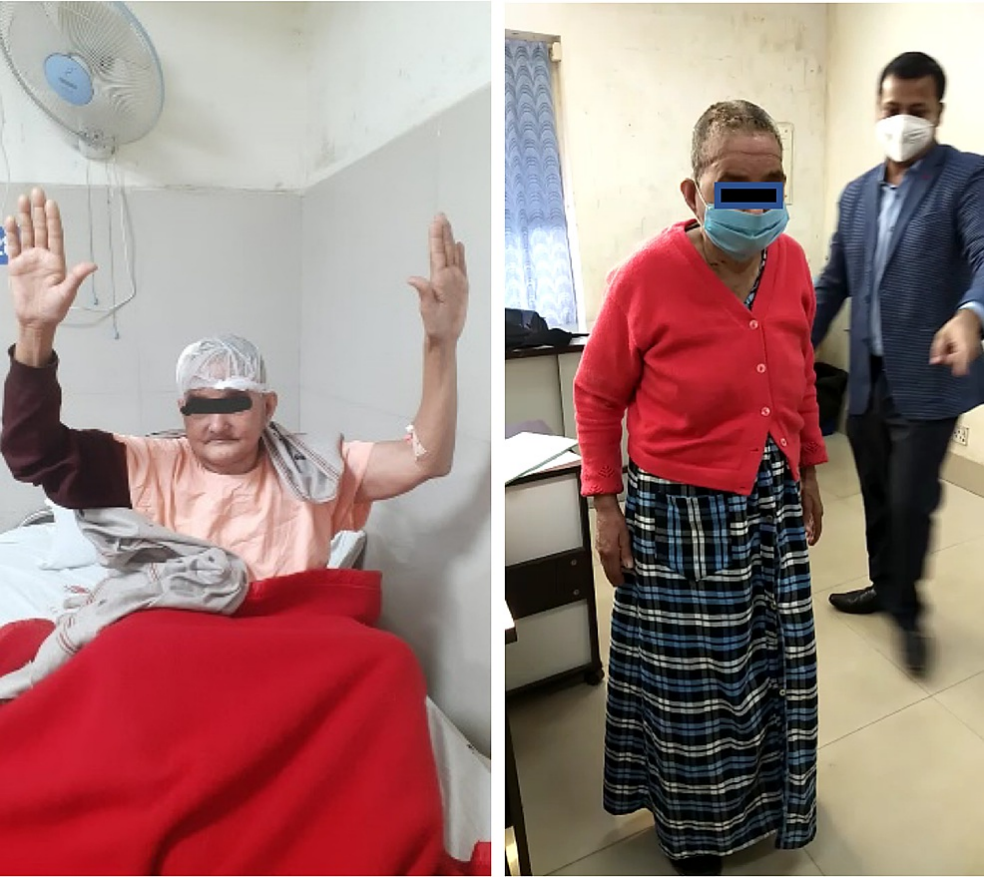
Patient at discharge and during follow-up.

The left parafalcine large tumor was excised. Part of the tumor invading the superior sagittal sinus was left behind (Simpson grade 3 excision) (Figure 3). She made a good post-op recovery, and her right-sided weakness had improved. She was able to walk without support by the time she was discharged on day 7 (Figure 4). Histopathological examination showed an atypical meningioma (WHO Grade 2). Adjuvant radiotherapy was advised for the part of the tumor left behind. Genetic testing was carried out for NF2, which was negative. She had no neurological deficits on her subsequent follow-ups at 1 and 3 months.

## Discussion

Meningiomas are among the most common intracranial neoplasms encountered by neurosurgeons in clinical practice. They arise from the arachnoid cap cells and are mostly benign. Their incidence ranges between 13 and 25% of all intracranial tumors [1]. Most of these tumors are sporadic and solitary.

Multiple meningiomas are uncommon and are usually associated with NF2. Multiple meningiomas in the absence of NF2 are rare, making our case unique. They have been defined as the presence of two or more lesions occurring simultaneously or sequentially at different locations [2]. In most cases, the lesions are located on the same side but sometimes may be bilateral [3].

The incidence of multiple meningiomas has been described as 2.3-8.9% of all intracranial meningiomas [4]. However, recent studies have reported the incidence on the higher side, mostly owing to the advancement and widespread availability of diagnostic infrastructure in terms of CT and MRI [4]. Similar to other meningiomas, multiple meningiomas have a significant female preponderance, and the incidence increases with age, the mean age of presentation being the sixth decade [5].

The associated risk factors are genetic predisposition, exposure to ionizing radiation, and hormones [6]. Our patient did not have any of these risk factors. The clinical features depend on the location of the tumors, the most common being headaches and seizures [7]. Since these are slow-growing tumors, many patients remain asymptomatic and may be diagnosed after neuroimaging is done due to unrelated reasons.

The treatment options include observation, surgery with or without adjuvant radiotherapy, and stereotactic radiosurgery [8]. Observation is a good option in patients with incidental small tumors with no surrounding edema. They would, however, require regular follow-ups and neuroimaging. Surgery is the treatment of choice in most patients, with the principles being the same as in solitary meningiomas [8]. In our case, while the large left parafalcine tumor was excised (Simpson Grade 3) [9], the left frontal and the left parafalcine small tumor were left behind for observation in view of their small size and lack of perilesional edema.

Adjuvant radiotherapy may be considered in patients who have had a higher Simpson Grade tumor removal, as was done in our case. However, stereotactic radiosurgery is reserved in cases where the tumor size is small [8].

## Conclusions

Multiple sporadic meningiomas in the absence of NF2 are very uncommon, with research still ongoing on its etiology. They are more common in females and usually present late in life, mean age being the sixth decade. Management is a challenge owing to the multiple lesions, especially if bilateral, where surgery may need to be staged. Surgery is the treatment of choice, with surgical principles being similar to solitary meningiomas. The overall outcome is good in multiple meningiomas due to the disease's benign nature. Like in solitary meningiomas, the recurrence rate depends on the Simpson Grade of tumor excision and the histological subtype.
